# Imaging of atherosclerosis with [^64^Cu]Cu-DOTA-TATE in a translational head-to-head comparison study with [^18^F]FDG, and Na[^18^F]F in rabbits

**DOI:** 10.1038/s41598-023-35302-5

**Published:** 2023-06-07

**Authors:** Constance E. Grandjean, Sune F. Pedersen, Camilla Christensen, Altea Dibenedetto, Thomas Eriksen, Tina Binderup, Andreas Kjaer

**Affiliations:** 1 Department of Clinical Physiology, Nuclear Medicine and PET & Cluster for Molecular Imaging, Copenhagen University Hospital–Rigshospitalet & Department of Biomedical Sciences, University of Copenhagen, Blegdamsvej 9, 2100 Copenhagen, Denmark; 2 Department of Veterinary Clinical and Animal Sciences, University of Copenhagen, Copenhagen, Denmark

**Keywords:** Atherosclerosis, Preclinical research

## Abstract

Atherosclerosis is a chronic inflammatory disease of the larger arteries that may lead to cardiovascular events. Identification of patients at highest risk of cardiovascular events is challenging, but molecular imaging using positron emission tomography (PET) may prove useful. The aim of this study was to evaluate and compare head-to-head three different PET tracers. Furthermore, tracer uptake is compared to gene expression alterations of the arterial vessel wall. Male New Zealand White rabbits (control group; n = 10, atherosclerotic group; n = 11) were used for the study. Vessel wall uptake was assessed with the three different PET tracers: [^18^F]FDG (inflammation), Na[^18^F]F (microcalcification), and [^64^Cu]Cu-DOTA-TATE (macrophages), using PET/computed tomography (CT). Tracer uptake was measured as standardized uptake value (SUV), and arteries from both groups were analyzed ex vivo by autoradiography, qPCR, histology, and immunohistochemistry. In rabbits, the atherosclerotic group showed significantly higher uptake of all three tracers compared to the control group [^18^F]FDG: SUV_mean_ 1.50 ± 0.11 versus 1.23 ± 0.09, *p* = 0.025; Na[^18^F]F: SUV_mean_ 1.54 ± 0.06 versus 1.18 ± 0.10, *p* = 0.006; and [^64^Cu]Cu-DOTA-TATE: SUV_mean_ 2.30 ± 0.27 versus 1.65 ± 0.16; *p* = 0.047. Of the 102 genes analyzed, 52 were differentially expressed in the atherosclerotic group compared to the control group and several genes correlated with tracer uptake. In conclusion, we demonstrated the diagnostic value of [^64^Cu]Cu-DOTA-TATE and Na[^18^F]F for identifying atherosclerosis in rabbits. The two PET tracers provided information distinct from that obtained with [^18^F]FDG. None of the three tracers correlated significantly to each other, but [^64^Cu]Cu-DOTA-TATE and Na[^18^F]F uptake both correlated with markers of inflammation. [^64^Cu]Cu-DOTA-TATE was higher in atherosclerotic rabbits compared to [^18^F]FDG and Na[^18^F]F.

## Introduction

Atherosclerosis is a chronic inflammatory disease of the arterial vessel wall, characterized by the accumulation of lipids and fibrous elements^1^. As one of the leading causes of cardiovascular morbidity and mortality, better treatments and optimized diagnostic tools are warranted.

In time, atherosclerosis may progress into vulnerable plaques ultimately leading to plaque rupture^2,3^. The vulnerable plaque is characterized by intraplaque hemorrhage, inflammatory cell infiltration, neovessel formation of the vasa vasorum, and development of a thin fibrous cap^4^. During the progression of atherosclerosis, calcification occurs. The inflammatory processes involved in the progression of atherosclerosis are essential targets for visualizing and treating atherosclerosis.

For functional characterization and treatment monitoring, non-invasive molecular imaging modalities are very useful. Molecular imaging is specialized to visualize specific cellular components or metabolic pathways. Positron emission tomography (PET) provides valuable information on different molecular processes^5^. The most commonly used PET tracer in a clinical setting is 2-[^18^F]fluoro-2-deoxy-D-glucose ([^18^F]FDG)^6^. It accumulates in metabolically active cells such as the vascular macrophages found in the inflamed plaque. [18F]FDG is useful to both quantify and track inflammation in atherosclerosis^7–11^. Unfortunately, the physiological uptake of [^18^F]FDG by cardiomyocytes challenges the visualization of inflammation in the coronary arteries^12^. These limitations can be eliminated using other PET tracers with lower myocardial uptake. The PET tracer [^64^Cu]Cu-DOTA-TATE detects somatostatin receptor 2 (SSTR2) expression on the surface of activated macrophages. Hence, it has been proven useful for assessing vascular inflammation^13–15^. Another PET tracer, Na[^18^F]F, has been used for characterization of early vascular vessel wall microcalcifications^16–18^. It is suggested that Na[^18^F]F uptake can serve as a non-invasive biomarker in risk stratification of increased cardiovascular patients^19^. The three PET tracers have separately been investigated. However, a head-to-head comparison of the tracers and correlation to the underlying gene expression alterations in the vessel wall, has to the best of our knowledge not been undertaken.

In this study, we aimed to assess and characterize key molecular components of the atherosclerotic process in a rabbit model. Three PET tracers targeting inflammation and microcalcification, were used and PET uptakes compared to gene expression alterations in the arterial vessel wall.

## Methods

All animal experiments were performed in accordance with appropriate approvals from the Animal Research Committee of the Danish Ministry of Justice (license number: 2016-15-0201-00831) and the study was conducted by following the ARRIVE and AVMA guidelines. All methods were performed in accordance with the relevant guidelines and regulations.

### Rabbits

**Figure 1:**
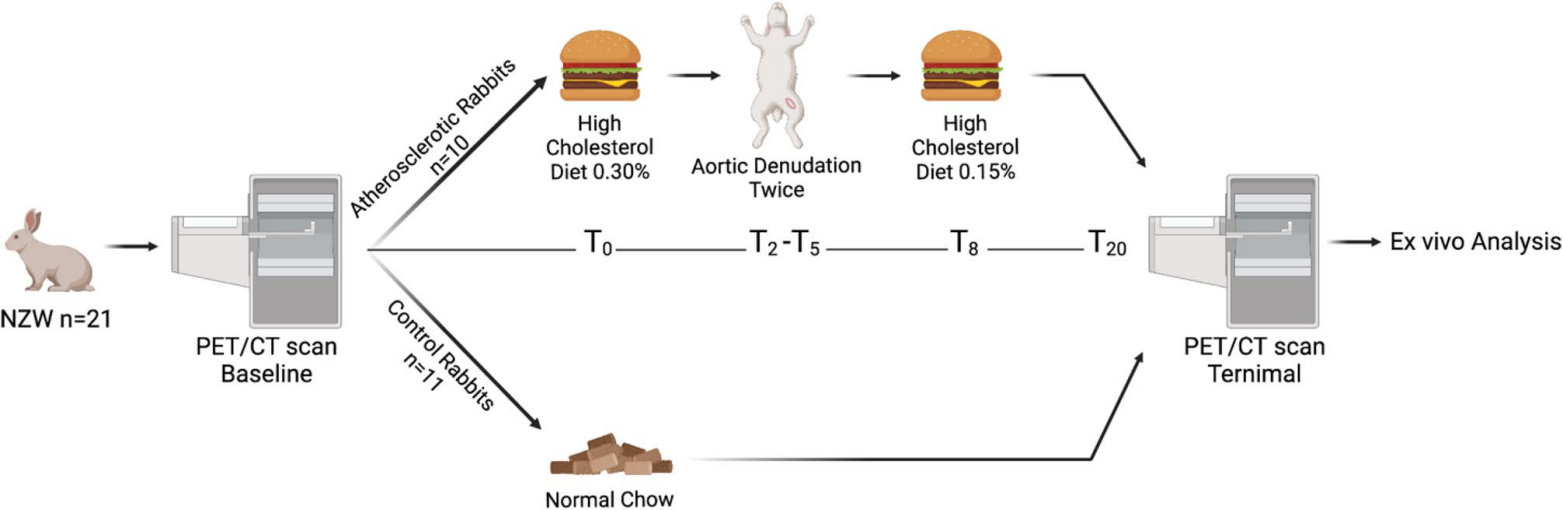
General characteristics of the control group and the atherosclerotic group. Study outline with both dietary (High Cholesterol Diet containing 0.30% cholesterol and 0.15% cholesterol) and surgical interventions for the atherosclerotic group together with scan times for both groups. T_0_ = study initiation, two weeks after (T_2_) the first surgical intervention was performed and five weeks after initiation (T_5_) the second surgical intervention was performed. After eight weeks (T_8_) the diet was switched from 0.30% HCD to 0.15% HCD. The study continued for another 16 weeks and after 20 weeks (T_20_) the study was finalized by PET/CT scans of the rabbits followed by ex vivo analyses.

Twenty-one male SPF New Zealand White rabbits (11 weeks old) were randomized into a control group (n = 10) and an atherosclerotic group (n = 11) (KB Lidköping Kaninfarm, Sweden). The rabbits were single housed. Atherosclerosis was induced by a combination of diet and denudation of the abdominal and thoracic aorta. The atherosclerotic group were fed a high cholesterol diet (HCD) containing 0.30% cholesterol, two weeks prior to the first surgery. After eight weeks and the remining study period, rabbits were shifted to 0.15% HCD. The control group was fed a normal chow diet (see study outline Fig. 1).

Under aseptic conditions, the atherosclerotic group underwent denudation by firstly localizing the femoral artery in one of the extremities. Then introducing a 4F-Fogarty embolectomy catheter into the artery before advancing the balloon approximately 20 cm. A repetitive action was performed by inflating the balloon and retracting it three times to ensure vascular injury. Finally, the muscle layers and skin were closed with absorbable and non-absorbable sutures, respectively. The procedure was repeated after three weeks on the contralateral extremity. Infection prophylaxis enrofloxacin (15 mg/kg) was injected intramuscularly before surgery.

Post-operative care consisted of the analgesic buprenorphine (0.01–0.05 mg/kg, subcutaneous injection (s.c.)) in combination with ketoprofen (3 mg/kg, intramuscular injection (i.m.)), administered on the day of surgery.

Euthanization of the rabbits was an initial sedation by an injection of ketamine (35 mg/kg) and xylazine (5 mg/kg) i.m. followed by a lethal injection of penobarbital (140 mg/kg) i.v. following the AVMA guidelines for the Euthanasia of Animals.

### Small animal PET imaging and image analysis

All the rabbits (n = 21) were baseline scanned on a dedicated small animal PET/Computed Tomography (CT) system (Inveon, Siemens Medical Systems, PA, USA) with the tracer [^18^F]FDG after which the rabbits were randomized into the two groups. Scans were performed as a one-bed position scan covering the abdominal aorta. The scans were performed 3 weeks prior to surgery of the atherosclerotic group and twenty weeks post-surgery, terminal scans were performed on both groups with different tracers: (1) [^18^F]FDG, (2) Na[^18^F]F, and (3) [^64^Cu]Cu-DOTA-TATE. All three tracers circulated for 60 min prior to PET imaging with an acquisition time of 1200 s. The radioactive dose for each tracer was: 123.4 ± 15.6 MBq ([^18^F]FDG), 116.0 ± 10.2 MBq (Na[^18^F]F), and 84.41 ± 5.78 MBq ([^64^Cu]Cu-DOTA-TATE). The 3 scans were performed in a randomized order to ensure ex vivo autoradiography of all 3 tracers for both groups. To allow physical decay of the tracers, a gap of at least 24 h following tracer injection with ^18^F-labeled compounds (physical half-life of ^18^F: 110 min) and 72 h following [^64^Cu]Cu-DOTA-TATE injection (physical half-life of ^64^Cu: 12.7 h) was ensured.

Images were analyzed as fused PET/CT images using the Inveon Research Workspace 4.2 software (Siemens Medical Systems, PA, USA) by drawing circular Regions of Interests (ROIs) on the abdominal aorta, from the iliac bifurcation to the right renal artery on every third slide. ROIs were drawn in the axial plane of CT images and superimposed on aligned PET images. One volume cylinder was created from all the ROIs drawn on the aorta, volume of interest (VOI). The mean Standardized Uptake Values (SUV_mean_) were calculated as the tracer uptake in the VOI divided by decay corrected injected activity and divided by the weight of the rabbit. Results are reported as the average SUV for the whole aortic segment (SUV_mean_) for best comparison to gene expression results. Tracer-to-Background Ratios (TBR) were obtained as the [^18^F]FDG uptake in the artery divided by the blood pool [^18^F]FDG uptake from the vein.

### Ex vivo analysis

Immediately following the last scan, rabbits were euthanized, and segments of the aorta were selected for specific ex vivo analysis. Autoradiography was performed for all three tracers with the aortic arch and half of the descending thoracic aorta (n = 4 for each tracer). The thoracic part of the aorta was chosen for autoradiography, because the tissue gets squeezed during exposure in the cassette, and we wanted to preserve the morphology in the abdominal part where PET/CT imaging was performed.

A lower part of the abdominal aorta above the iliac bifurcation was chosen for RNA extraction.

The thoracic part, the remaining part of the abdominal aorta, and the aortic arch were fixed in 4% formalin for 24 h before being sectioned into 2–3 mm pieces and embedded in paraffin. A series of axial sections (4 µm thickness) were obtained and selected for histology and immunohistochemistry (IHC).

For general morphological characterization, a Hematoxylin & Eosin (H&E) stain was performed and a Von Kossa stain for calcifications.

For IHC, RAM 11 (Monoclonal Mouse Anti-Rabbit Macrophage, Clone RAM11, Agilent DAKO, USA) was used for assessing macrophage infiltration. Envision FLEX DAB + Substrate Chromogen System (Agilent, DAKO, USA) was used for exposing immunoreactivity together with hematoxylin as counterstain.

### Real-time reverse transcription—polymerase chain reaction

The aortas were immediately after extraction stored in RNAlater Stabilization Solution (ThermoFisher, MA, USA) for 24 h at 5 °C before removed from the solution and stored at − 80 °C until further use. All reagents and kits were purchased from QIAGEN (Hilden, Germany). Total RNA was extracted with TRIzol® Reagent and reverse-transcribed into cDNA using the RT^2^ First Strand Kit. Two different arrays were used: Rabbit wound healing array (QIAGEN, PANZ-121ZA-RT^2^ Profiler PCR Array) and a custom array (QIAGEN, CLAN32799A—Custom RT^2^ PCR Array). Plates were read on the Mx3000P real-time PCR system (Stratagene, CA, USA). The results were analyzed using the online software GeneGlobe (QIAGEN). Gene expression levels of genes-of-interest (GOI) were normalized to the level of the reference genes ACTA2, ACTB, GAPDH, LDHA, and non-POU domain containing octamer-binding-like (LOC100346936). The data was analyzed using the 2-deltaCt method.

### Statistical analysis

Statistical analysis of the in vivo data was obtained in GraphPad Prism version 8 (GraphPad Software Inc., USA).

An unpaired two-tailed t-test was performed to compare the tracer uptake between groups at the different time points and compare fold regulation of genes. Correlation between gene expression levels and tracers were analyzed using a Pearson’s correlation. *P-*values less than 0.05 were considered significant.

All data are presented as mean ± Standard Error of the Mean (SEM).

## Results

The characteristic differences between the atherosclerotic and control groups were assessed by molecular imaging, histology, IHC, and gene expression analyses.

**Figure 2:**
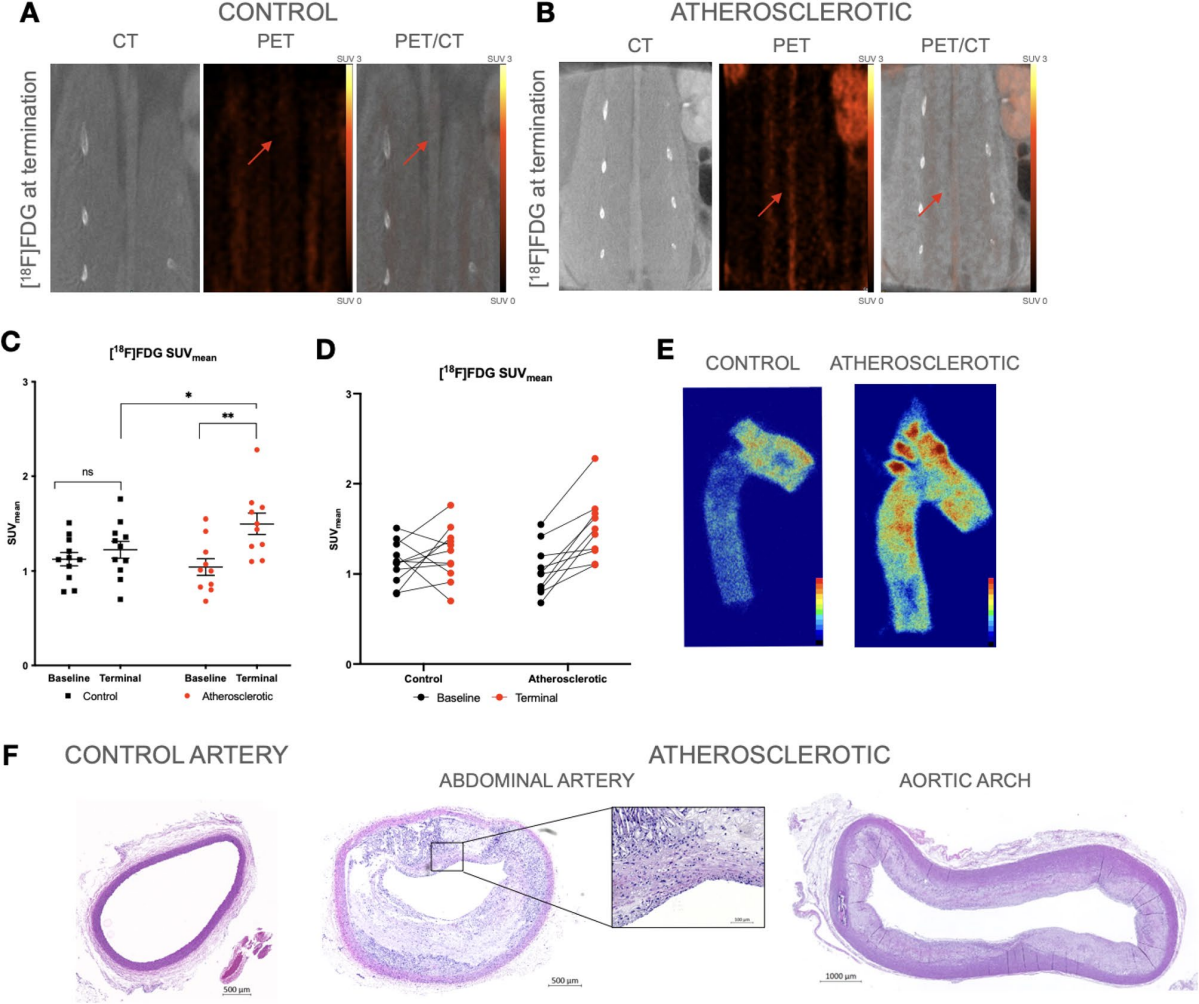
[^18^F]FDG in vivo PET/CT Imaging and ex vivo analysis. (**A**) CT, PET, and fused PET/CT images in the coronal plane, from a control rabbit with uptake of [^18^F]FDG. The control rabbits show a homogenous uptake of [^18^F]FDG (red arrow marking the artery on the PET and PET/CT images). (**B**) CT, PET, and fused PET/CT images in the coronal plane of [^18^F]FDG uptake in an atherosclerotic rabbit. The uptake is clearly visualized throughout the abdominal artery on both the PET and PET/CT images marked with the red arrows. Color bars are calibrated in SUV and with no background subtracted. Color bars are identical for the two groups. (**C**) Dot plots show in vivo SUV_mean_ values ± SEM from baseline and terminal scans of [^18^F]FDG. The baseline scan shows no significant (ns) difference between the groups. The control group (n = 10) from baseline to terminal scan showed no significant difference, while for the atherosclerotic group (n = 11) there was a significant difference in [^18^F]FDG uptake (**), *p* = 0.0029. The terminal scans reveal a significant (*) difference in [^18^F]FDG uptake between the atherosclerotic group and the control group (SUV_mean_ 1.50 ± 0.112 vs SUV_mean_ 1.23 ± 0.089, *p* = 0.025). (**D**) Spaghetti plots showing the change in [^18^F]FDG uptake from baseline to end of study for each rabbit of both groups. (**E**) Autoradiographic images show the binding of [^18^F]FDG in the aortic arch and descending thoracic artery from a control and an atherosclerotic artery. (**F**) Cross section H&E stains of the abdominal artery and aortic arch. The atherosclerotic artery show wall thickening and plaque formation causing a narrowed lumen of the artery compared to the lumen of the normal artery. The aortic arch show similar morphological changes as the abdominal artery with present atherosclerosis.

At baseline, [^18^F]FDG PET/CT scans revealed no difference in uptake between the control group and the atherosclerotic group (SUV_mean_ 1.13 ± 0.07 vs SUV_mean_ 1.04 ± 0.088; *p* = 0.9). While [^18^F]FDG significantly increased from baseline to termination in the atherosclerotic group (*p* = 0.005)*,* only a small and non-significant increase was observed in the age-matched control group during the 20 weeks study period (*p* = 0.389)(Fig. 2D)*.* General characteristics for the atherosclerotic development in the arteries were established by histology (Fig. 2F).

### Assessment of metabolic activity in plaque through [^18^F]FDG PET imaging

At termination of the study, the [^18^F]FDG uptake seen in CT, PET and fused PET/CT images was evidently higher in the atherosclerotic group compared to the control group (Fig. 2A,B). We observed a significantly higher uptake of [^18^F]FDG in the atherosclerotic group compared to the control group (SUV_mean_ 1.50 ± 0.112 vs 1.23 ± 0.089; *p* = 0.025; Fig. 2C,D). The atherosclerotic group had a TBR_mean_ of 1.11 ± 0.1 and a TBR_max_ of 2.20 ± 0.1, while the control group had a TBR_mean_ of 0.99 ± 0.1 and a TBR_max_ of 2.14 ± 0.1.

Higher accumulation of [^18^F]FDG was also observed in the aortic arch of the atherosclerotic group by autoradiography (Fig. 2E). Accumulation of foam cells, cholesterol crystals, and narrowed lumens were found in the atherosclerotic arteries by H&E. The control group showed a thin and intact arterial wall (Fig. 2F).

### Assessment of microcalcification through Na[^18^F]F PET imaging

**Figure 3:**
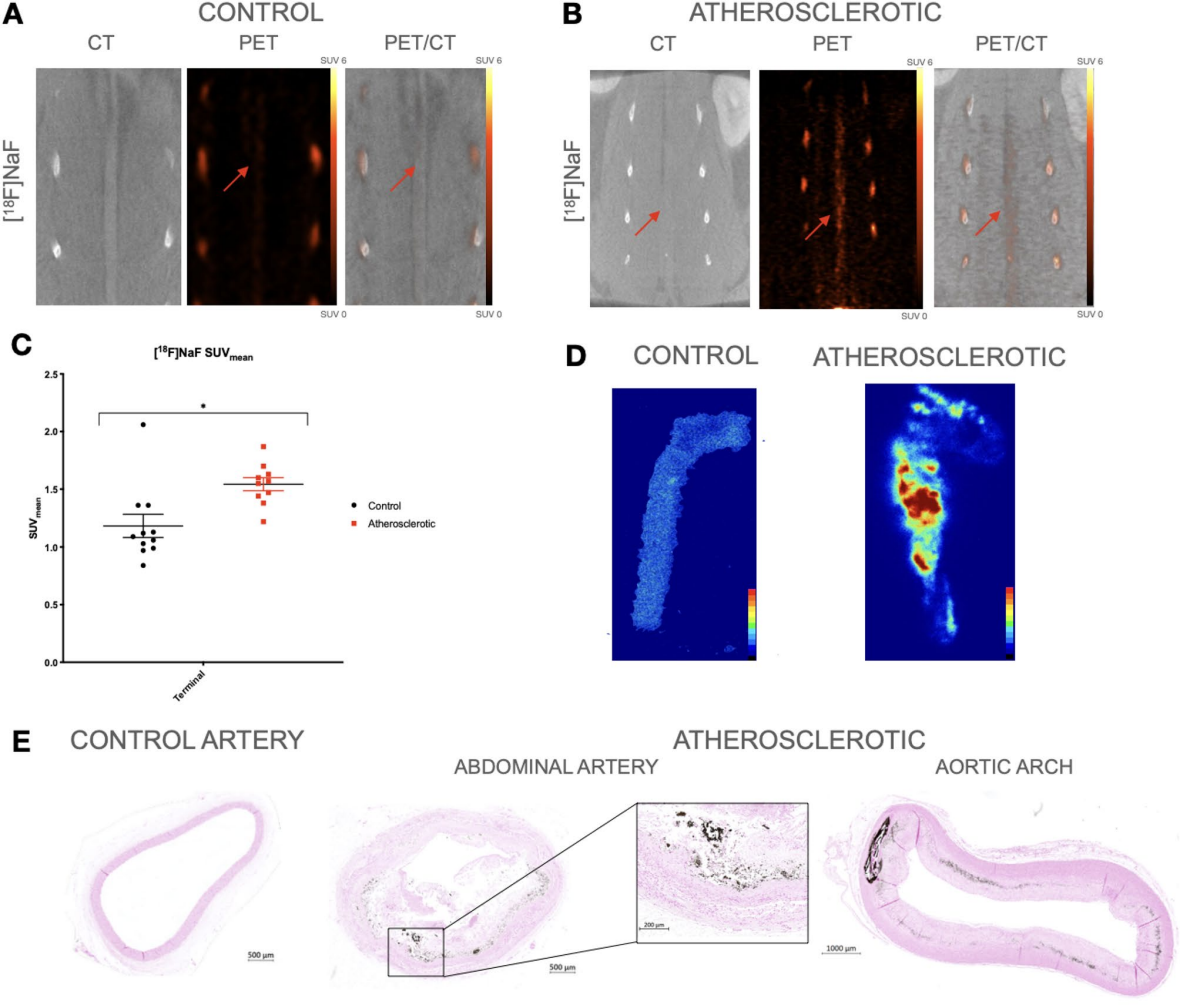
[^18^F]NaF in vivo PET/CT imaging and ex vivo analysis. (**A**) CT, PET, and fused PET/CT images in the coronal plane, from a control rabbit. The control rabbits show a homogenous uptake of [^18^F]NaF marked with red arrows on the PET and PET/CT. (**B**) An atherosclerotic rabbit CT, PET, and fused PET/CT images showed higher uptake of [^18^F]NaF. [^18^F]NaF showed specific higher uptake in calcified lesions of the abdominal artery, which can be seen on the CT, PET and PET/CT marked with a red arrow on each image. Color bars are calibrated in SUV with no background subtracted. Same scale bar used on both images. (**C**) SUV_mean_ values ± SEM of terminal [^18^F]NaF scans of the control group and atherosclerotic group presented by dot plots. The atherosclerotic group had a significantly higher uptake compared to the control group (SUV_mean_ 1.54 ± 0.057 vs SUV_mean_ 1.18 ± 0.099, *p* = 0.006). (**D**) Phosphor autoradiographic images show the binding of [^18^F]NaF in the aortic arch and descending thoracic artery from a control and an atherosclerotic artery. (**E**) Cross section of the abdominal artery stained with Von Kossa. No calcium deposits were evident, since none of the control arteries stained positive for Von Kossa. The Von Kossa stain of the atherosclerotic abdominal artery and the aortic arch show calcium deposits (black).

For assessment of microcalcifications within the arterial vessel wall, rabbits were scanned with Na[^18^F]F PET/CT(Fig. 3A,B). A significant difference in uptake was observed between the atherosclerotic group and the control group (SUV_mean_ 1.54 ± 0.057 vs SUV_mean_ 1.18 ± 0.099; *p* = 0.006; Fig. 3C). A heterogeneous and high accumulation of Na[^18^F]F was observed in the atherosclerotic group by autoradiography (Fig. 3D), confirming the PET findings. The presence and location of microcalcifications within the plaques are also shown by the Von Kossa staining (Fig. 3E).

### Assessment of inflammation through [^64^Cu]Cu-DOTA-TATE PET imaging

**Figure 4:**
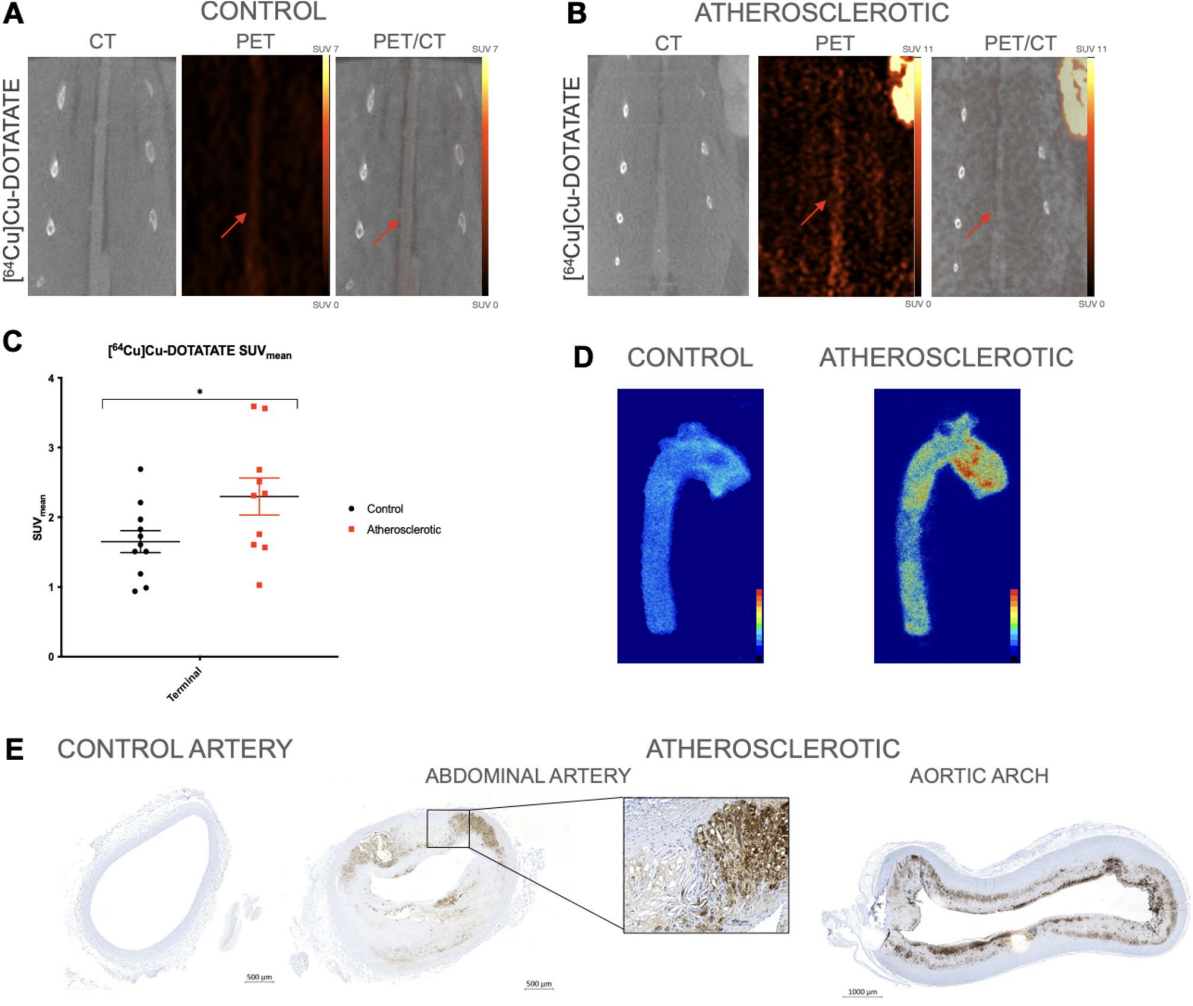
[^64^Cu]Cu-DOTA-TATE PET/CT imaging and ex vivo analysis. (**A**) CT, PET, and fused PET/CT images in the coronal plane, from a control rabbit with uptake of [^64^Cu]Cu-DOTA-TATE. The artery is marked with a red arrow. (**B**) CT, PET, and fused PET/CT images of an atherosclerotic rabbit with uptake of [^64^Cu]Cu-DOTA-TATE showed a high and homogenous uptake in the artery marked with the red arrow in both images. Color bars were calibrated in SUV with no background subtracted. Same scale bar used for both images. (**C**) Dot plots showed in vivo SUV_mean_ values ± SEM of the terminal scan of [^64^Cu]Cu-DOTA-TATE. The dot plot showed a significant (*) difference in [^64^Cu]Cu-DOTA-TATE uptake between the atherosclerotic group and the control group (SUV_mean_ 2.30 ± 0.266 vs SUV_mean_ 1.65 ± 0.157, *p* = 0.047). (**D**) Autoradiographic images show the binding of [^64^Cu]Cu-DOTA-TATE in the aortic arch and descending aorta from a control and an atherosclerotic artery. The binding of [^64^Cu]Cu-DOTA-TATE was higher in the atherosclerotic artery than in the control artery. (**E**) Cross section of the abdominal artery. The arteries were embedded in paraffin and IHC with was performed. The anti-rabbit macrophage (RAM11) staining shows macrophages surrounding the cholesterol crystals in the plaque in both the abdominal part and the aortic arch of the atherosclerotic artery.

In line with the findings of the other 2 tracers we found that [^64^Cu]Cu-DOTA-TATE uptake was significantly higher for the atherosclerotic group compared to the control group (SUV_mean_ 2.30 ± 0.266 vs SUV_mean_ 1.65 ± 0.157; *p* = 0.047), which was also observed in the PET and PET/CT images (Fig. 4A–C). Accumulation of [^64^Cu]Cu-DOTA-TATE in the arteries of the atherosclerotic group was confirmed by autoradiography (Fig. 4D). Abundant macrophage infiltration within the plaques was evident from the RAM11 stain. For the control arteries no presence of macrophages was observed (Fig. 4E) confirming the low uptake observed on PET and autoradiography.

### Head-to-head comparison of tracer uptake

**Figure 5:**
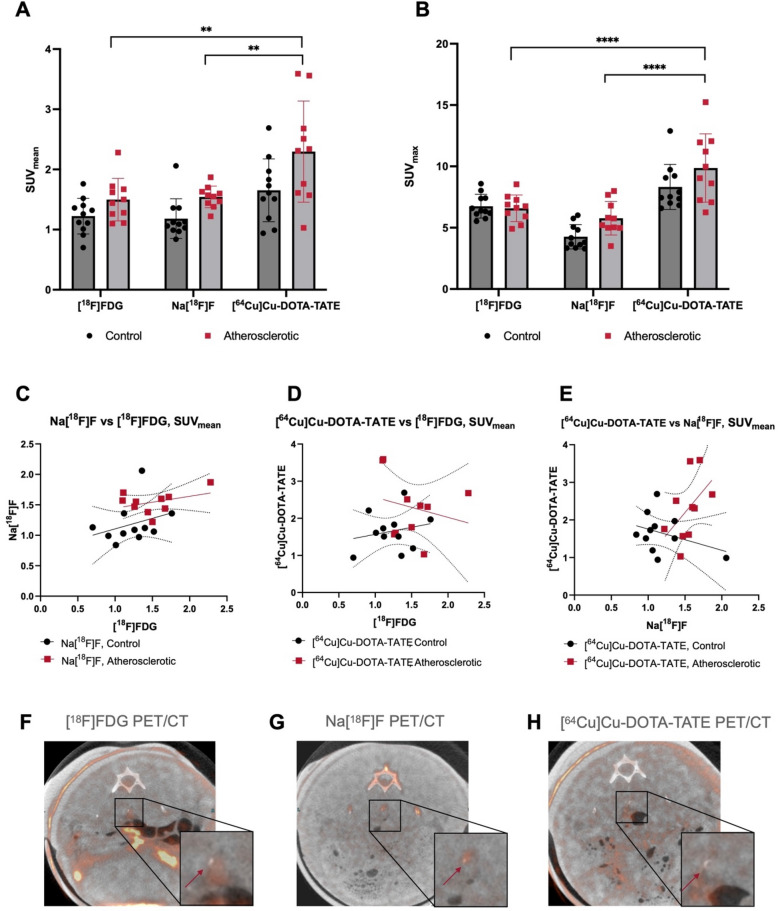
Head-to-head comparison of the three PET tracers together with corresponding PET/CT images. Grouped comparison of the three tracers with significantly higher uptake of [^64^Cu]Cu-DOTA-TATE compared to Na[^18^F] and [^64^Cu]Cu-DOTA-TATE compared to [^18^F]FDG for (**A**) SUVmean and (**B**) SUVmax. Correlation plots between (**C**) [^18^F]FDG and Na[^18^F]F, (**D**) [^18^F]FDG and [^64^Cu]Cu-DOTA-TATE and (**E**) between Na[^18^F]F and [^64^Cu]Cu-DOTA-TATE. (**F**) [^18^F]FDG PET/CT of atherosclerotic artery (red arrow). (**G**) Na[^18^F]F PET/CT in the same lesion (red arrow) and (**H**) [^64^Cu]Cu-DOTA-TATE uptake in the same area (red arrow).

In a head-to-head comparison of the three tracers, the [^64^Cu]Cu-DOTA-TATE tracer had the highest absolute uptake in the atherosclerotic group (Fig. 5A,B) and the largest difference between the atherosclerotic group and the control group (ratio-_SUVmean_ = 1.38) compared to [^18^F]FDG (ratio-_SUVmean_ = 1.22) and Na[^18^F]F (ratio-_SUVmean_ = 1.30). Pairwise analysis of the three tracers for the atherosclerotic group revealed a significantly higher uptake of [^64^Cu]Cu-DOTA-TATE compared to [^18^F]FDG (SUV_mean_: 2.29 vs 1.50, *p* = 0.0012 and SUV_max_: 9.86 vs 6.59, *p* < 0.0001) and when compared to Na[^18^F]F (SUV_mean_: 2.29 vs 1.55, *p* = 0.0027 and SUV_max_: 9.86 vs 5.79, *p* < 0.0001). For the control group Na[^18^F]F uptake was significantly lower than both [^18^F]FDG and [^64^Cu]Cu-DOTA-TATE when assessed as SUV_max_ (4.27 vs 6.75 (*p* = 0.0019) and 8.32 respectively (*p* < 0.0001)) whereas no significant difference was found for any of the three tracers in the control group when assessed as SUV_mean_.

By Pearson correlation analysis none of the tracers correlated significantly, with *r* = *0.381*, (*p* = 0.278) for the atherosclerotic group between [^18^F]FDG and Na[^18^F]F (Fig. 5C), *r* = *-0.227* (*p* = 0.529) for the atherosclerotic group between [^18^F]FDG and [^64^Cu]Cu-DOTA-TATE (Fig. 5D) and r = 0.503 (*p* = 0.138) for atherosclerotic group between Na[^18^F]F and [^64^Cu]Cu-DOTA-TATE (Fig. 5E). Differences in tracer accumulation are shown in Fig. 5F–H for the abdominal aorta of an atherosclerotic rabbit.

### Transcriptome analysis and correlation with imaging results

**Figure 6:**
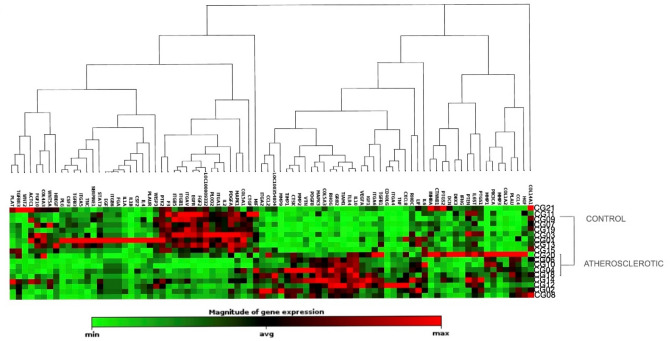
Gene Expression of the control group and the atherosclerotic group. Clustergram showing gene expression in the control group and the atherosclerotic group. Each group shows a specific cluster of highly expressed genes compared to the other group.

Clustergram analysis separated the atherosclerotic rabbits from the control rabbits and identified two clusters with differentially expressed genes (Fig. 6). Of the 102 GOIs analyzed, 34 GOIs were significantly upregulated in the atherosclerotic group compared to the control group, and 18 GOIs were significantly downregulated in the atherosclerotic group compared to the control group. Generally, genes associated with cell surface receptors, extracellular matrix remodeling, and inflammatory cytokines and chemokines were upregulated, whereas the downregulated genes were associated with cell adhesion, and vessel wall atherogenic effects (GeneGlobe, QIAGEN). The most differentially expressed genes were considered in the analysis of correlation with the three PET tracers, irrespectively of the two groups (Table 1).

**Table 1:** Correlation between GOIs and PET tracer.

	[^18^F]FDG		Na[^18^F]F		[^64^Cu]Cu-DOTA-TATE	
	Correlation coefficient	*p*-value	Correlation coefficient	*p*-value	Correlation coefficient	*p*-value
CD36			0.71	0.0003		
CD86			0.55	0.0094	0.49	0.0236
CD163			0.45	0.0033		
CTSK			0.49	0.0409	0.50	0.0332
GRB2	0.60	0.008	0.66	0.0029	0.55	0.0174
IL2					− 0.53	0.0235
IL10					0.54	0.0212
ITGAL			0.54	0.0113	0.47	0.0321
Transgelin			− 0.58	0.0121	− 0.65	0.0035
SCARF1			0.43	0.0498	0.45	0.0397
TGFB3			− 0.72	0.0007	− 0.56	0.0166
TIAM1			0.55	0.0183	0.56	0.0168
TNF	0.48	0.0446	0.48	0.0453		

Correlation coefficient for each GOI and the three PET tracers, with corresponding *p*-values. CTSK, Cathepsin K; GRB2, Growth Factor receptor-bound protein 2; IL-2, Interleukin 2; IL-10, interleukin 10; ITGAL, Integrin Alpha L; SCARF1, Scavenger receptor class f member 1; TGFB3, Transforming growth factor; beta 3; TIAM1, T-cell lymphoma invasion and metastasis 1; TLR4, Toll-like receptor 4; TNF, Tumor Necrosis Factor.

A few of the analyzed genes correlated with the uptake of [^18^F]FDG. However, most of the correlations were found with Na[^18^F]F and [^64^Cu]Cu-DOTA-TATE (Table 1).

Cathepsin K (CTSK) showed a 5.40-fold upregulation (*p* < 0.00001) in the atherosclerotic group compared to the control group together with positive correlations with both Na[^18^F]F (r = 0.49, *p* = 0.0409) and [^64^Cu]Cu-DOTA-TATE (r = 0.50, *p* = 0.0332) (see Table 1). The same trend was seen with the macrophage marker, CD86, displaying a 78-fold upregulation in the atherosclerotic group compared to the control group (*p* < 0.00001) (see Table 2). A 235-fold upregulation of integrin alpha L (ITGAL) was found in the atherosclerotic group compared to the control group as well as a positive correlation with Na[^18^F]F (r = 0.54, *p* = 0.0113) and [^64^Cu]Cu-DOTA-TATE (r = 0.47, *p* = 0.0321).

**Table 2:** Fold upregulation in atherosclerotic group compared to control group.

	Fold upregulation	*p*-value
CD163	4.48	0.00218
CD36	13.70	< 0.00001
CD86	78.00	< 0.00001
CTSK	5.40	< 0.00001
ITGAL	235.00	< 0.00001
SCARF1	5.60	< 0.00001
TIAM1	29.90	< 0.00001
TNF	4.06	0.00251

CTSK, Cathepsin K; ITGAL, Integrin Alpha L; SCARF1, Scavenger receptor class f member 1; TIAM1, T-cell lymphoma invasion and metastasis 1; TNF, Tumor Necrosis Factor.

## Discussion

In this study, we demonstrated the usefulness of [^64^Cu]Cu-DOTA-TATE for assessing atherosclerosis in a rabbit model. We showed by PET/CT and autoradiography that [^64^Cu]Cu-DOTA-TATE, [^18^F]FDG, and Na[^18^F]F accumulate in atherosclerotic plaques. Our imaging results support our findings of pronounced morphological alterations in the arterial vessel wall and the upregulation of pro-atherogenic molecular markers in the atherosclerotic arteries.

[^18^F]FDG has been the common tracer used for PET imaging of atherosclerosis due to its ability to visualize metabolic activity in cells^14^. [^18^F]FDG uptake is associated with macrophage differentiation, cell activation and cellular glucose metabolism^5,20,21^. However, the high uptake of [^18^F]FDG in healthy myocardium and thus spillover limits its use in the coronary arteries.

[^64^Cu]Cu-DOTA-TATE specifically targets cells expressing SSTR2. Since SSTR2 have been found on the surface of activated macrophages and at high levels in atherosclerotic plaques it provides an attractive alternative to [^18^F]FDG with its low uptake in healthy myocardium and a higher uptake of [^64^Cu]Cu-DOTA-TATE in macrophages when compared to [^18^F]FDG ^12^. In the current study we also found a significantly higher [^64^Cu]Cu-DOTA-TATE uptake compared to both [^18^F]FDG and Na[^18^F]F. While both [^18^F]FDG and [^64^Cu]Cu-DOTA-TATE targets inflammation, we were not able to find a correlation between tracer uptake for any of the 3 tracers investigated, pointing towards distinct information achieved by each tracer.

A few clinical studies have reported successful use of [^68^Ga]Ga-DOTA-TATE in assessing the atherosclerotic burden in patients^14,22,23^. In the present study, we used [^64^Cu]Cu-DOTA-TATE, which binds to the same target but due to a four-fold lower positron range of [^64^Cu] compared to [^68^Ga] (≈1 mm vs 4 mm), [^64^Cu] provides a much better spatial resolution of particular value when evaluating small structures as the vessel wall. We previously demonstrated the beneficial effect of [^64^Cu] compared to [^68^Ga] in vascular imaging with higher uptake and better correlation to cardiovascular risk factors^24^. In the current study we found a higher uptake of [^64^Cu]Cu-DOTA-TATE compared to a recent study in the same model where [^68^Ga]Ga-DOTA-TATE was used^12^.

Following plaque inflammation, osteogenic differentiation gives rise to microcalcification development driven by mineral metabolic activity. Microcalcifications are markers of more unstable stages of atherosclerosis, which could potentially lead to plaque rupture caused by destabilization of the fibrous cap surrounding the plaque^25^. Macrocalcifications can be detected by CT at late stages unlike microcalcifications which are not detectable by CT^26^. The uptake of Na[^18^F]F in early microcalcifications is correlated to the healing response of atherosclerotic plaques. Thus Na[^18^F]F is associated with inflammation and high-risk plaque^27^. In this study, the atherosclerotic arteries showed significantly higher uptake of Na[^18^F]F compared to age-matched controls, possibly indicating metabolically active mineralization. Figure 3B shows particular lesions with high uptake of Na[^18^F]F, which confirm the presence of microcalcifications and atherosclerosis, while no calcifications were evident on the CT scans of the atherosclerotic rabbits. We validated the PET/CT findings with histology and IHC. The stains confirmed the infiltration of macrophages surrounding the foam cells and cholesterol crystals, and the presence of microcalcifications. Autoradiographic findings from the thoracic aorta were also confirmed by histology and IHC, and no pronounced difference in atherosclerotic burden was found between the abdominal and thoracic part of the aorta.

Comparing the gene expression of atherosclerotic arteries to control arteries revealed upregulated genes associated with atherosclerosis through various pathways. The most upregulated genes were chosen for a correlation analysis with the three tracers used in this study. Only a few of the genes correlated with [^18^F]FDG, whereas several genes correlated with [^64^Cu]-Cu-DOTA-TATE and Na[^18^F]F accumulation. Previously, CTSK have been found to be associated with FDG-uptake in carotid plaque in humans and not associated with [^64^Cu]-Cu-DOTA-TATE uptake^28^. Contrarily, our findings show a positive correlation between CTSK and [^64^Cu]-Cu-DOTA-TATE and Na[^18^F]F, yet no correlation with [^18^F]FDG. We confirmed that [^64^Cu]-Cu-DOTA-TATE correlates to macrophage infiltration with a positive correlation with CD86but did not find a correlation with the marker of activated macrophages CD163 as previously found by our group^15^. A positive correlation was found between the macrophage markers CD36, CD86, and CD163 and Na[^18^F]F, indicating Na[^18^F]F activity correlates not only with microcalcification, but also macrophage infiltration^29^.

TNF is an activated M1 macrophage related cytokine and is involved in systemic inflammation. In our study, we found TNF to positively correlate with both [^18^F]FDG and Na[^18^F]F. These results are in line with a previous study finding a positive correlation in inflamed atherosclerotic plaques between TNF and [^18^F]FDG^30^.

IL-10 is an anti-inflammatory cytokine with deactivating properties in macrophage modulation and protects against atherosclerosis. It was upregulated in the atherosclerotic group by 14-fold and positively correlated with [^64^Cu]-Cu-DOTA-TATE. A predictor of atherosclerosis progression, ITGAL, was also found to correlate with both [^64^Cu]-Cu-DOTA-TATE and Na[^18^F]F. Of all the genes investigated, ITGAL was most differentially expressed between the atherosclerotic group and the control group. ITGAL expression is a signature for inflammatory monocytes, which together with other immune cells accumulates and become atherosclerotic plaque^31,32^.

## Perspective and limitations

The gene expression is performed ex vivo and demands an invasive approach. Molecular imaging by PET/CT provides a non-invasive approach. Furthermore, PET/CT scans can be performed at various time points to follow disease progression.

A limitation of the rabbit model is the lack of disease progression into unstable plaques and cardiovascular events. Intraplaque hemorrhage, plaque rupture, and infarctions are rarely seen in this model. Another limitation is the use of only male rabbits in the study; however, this was chosen to avoid the potential effect of hormonal changes in female rabbits. Future prospective clinical trials are needed to confirm that both [^64^Cu]Cu-DOTA-TATE and Na[^18^F]F predicts plaque vulnerability.

## Conclusion

We demonstrated the value of [^64^Cu]Cu-DOTA-TATE and Na[^18^F]F for assessment of atherosclerosis. The results obtained are distinct from those obtained with [^18^F]FDG and no significant correlations were found between the three tracers. [^64^Cu]Cu-DOTA-TATE uptake was significantly higher in atherosclerotic rabbits compared to both [^18^F]FDG and Na[^18^F]F. Key inflammatory biomarkers, such as CD36, CD86 and ITGAL, were upregulated in the atherosclerotic rabbits and correlated with tracer accumulation of [^64^Cu]Cu-DOTA-TATE and Na[^18^F]F but did not correlate with [^18^F]FDG.

## Data Availability

The datasets generated and analyzed during the current study are available in the GEO repository, https://www.ncbi.nlm.nih.gov/geo/query/acc.cgi?acc=GSE220754.
